# Feasibility of Treating High Grade Gliomas in Children with Tumor-Treating Fields: A Case Series

**DOI:** 10.7759/cureus.10804

**Published:** 2020-10-05

**Authors:** John Crawford, Marlon G Saria, Girish Dhall, Ashley Margol, Santosh Kesari

**Affiliations:** 1 Neurosciences and Pediatrics, University of California San Diego, San Diego, USA; 2 Medicine, Pacific Neuroscience Institute, John Wayne Cancer Institute at Providence Saint John’s Health Center, Santa Monica, USA; 3 Pediatric Hematology, Oncology, and Marrow Transplantation, University of Alabama at Birmingham, Birmingham, USA; 4 Medicine, Children's Center for Cancer and Blood Diseases, Children’s Hospital of Los Angeles, Los Angeles, USA

**Keywords:** high grade gliomas, tumor treating fields, pediatric, neuro-oncology

## Abstract

Children diagnosed with high grade gliomas (HGG) have dismal prognoses and treatment options remain limited. Tumor treating fields (TTFields) in combination with temozolomide (TMZ) is approved for the treatment of newly diagnosed and recurrent glioblastoma (GBM) in adult patients. However, clinical experience with TTFields in the pediatric HGG population is lacking.

This retrospective review of four clinical cases was undertaken to evaluate the feasibility of treating children diagnosed with HGG off-label with TTFields. Patients were evaluated for device compliance, safety, and outcome. Treatment with TTFields was delivered via four transducer arrays placed on the shaved scalp, which were connected to a portable device generating 200 kHz alternating electric fields.

One female and three male patients (ages 4-16 years) with heavily pretreated HGG were treated with TTFields off-label from March 2015 to December 2016. In three of these cases, TTFields were administered in combination with TMZ. Across all four patient cases, average wear compliance rates ranged between 53% and 92%. No device-related toxicities were reported during treatment with TTFields delivered for up to four months. All patients eventually died of the disease.

TTFields was well tolerated in our limited cohort of patients. Compliance times were similar to what has been reported in adults without significant toxicity. Further studies of the efficacy and safety of TTFields in children with HGG are underway in a clinical trial setting.

## Introduction

Glioblastoma (GBM) is a highly malignant brain tumor typically diagnosed in older adults (median age 64 years) and is very rare in children [1-2]. In the US population, over the period 2009-2013, only 1.25% of all cases of GBM occurred in those aged under 20 years; GBM represented only 2.9% of central nervous system tumors reported in children up to 19 years of age [3]. Similar to adult patients with GBM, the prognosis in children with GBM is generally very poor. Median survival time for adults with GBM has been reported as only 14.6 months [4], although there is some evidence that younger patients survive slightly longer, perhaps related to better performance status [5]. Recent US data showed a two-year survival of 33.3% and a five-year survival of 16.8% in patients with GBM below 20 years of age compared with 16.9% and 5.5%, respectively, across all age groups [3].

Prior to tumor treating fields (TTFields), treatment recommendations for adult patients with GBM (up to 70 years of age) involved a multidisciplinary approach of maximum safe surgical resection, radiotherapy, and concurrent and adjuvant chemotherapy [6]. The addition of the oral alkylating agent temozolomide (TMZ) to radiation treatment has been shown to prolong survival by 15-17 months in adult patients with GBM (two-year overall survival [OS] rate of 26% in adults with GBM vs 10% for radiation therapy alone) [4,6-7]. In pediatric high grade gliomas (HGG), TMZ has failed to show improved outcomes in the clinical trial setting [8].

TTFields are non-invasive, low-intensity, intermediate frequency (200 kHz), alternating electric fields, delivered in a loco-regional manner. The inhibitory effect of TTFields on cell growth is mainly mediated by interference with the mitotic cell cycle, in particular during metaphase, anaphase, and telophase [9-10].

Treatment with TTFields in combination with TMZ maintenance therapy has been shown to significantly prolong survival and is well tolerated in adults with newly diagnosed GBM [11]. TTFields has also been shown to be as effective as chemotherapy in adults with recurrent GBM and is associated with improved quality of life [12]. Based on these trial results, TTFields therapy was approved for adults with recurrent and newly diagnosed GBM, but is currently not approved for use in pediatric patients. Until more recently, there was no reported experience of TTFields in pediatric GBM. A single case study reported the benefits of TTFields therapy combined with bevacizumab in one pediatric patient with GBM in whom TTFields treatment was associated with stable disease for seven months with minimal adverse events [13]. A case series of TTFields in five pediatric patients (ages 10-20 years) with high-grade gliomas showed that the treatment was well tolerated without treatment-limiting toxicities [14]. Here we review our retrospective series of four patients diagnosed with HGG treated with TTFields off-label, including the two youngest patients reported. Our limited experience regarding the safety and tolerability of TTFields, even in young children, is encouraging.

## Case presentation

Case 1

**Figure 1:**
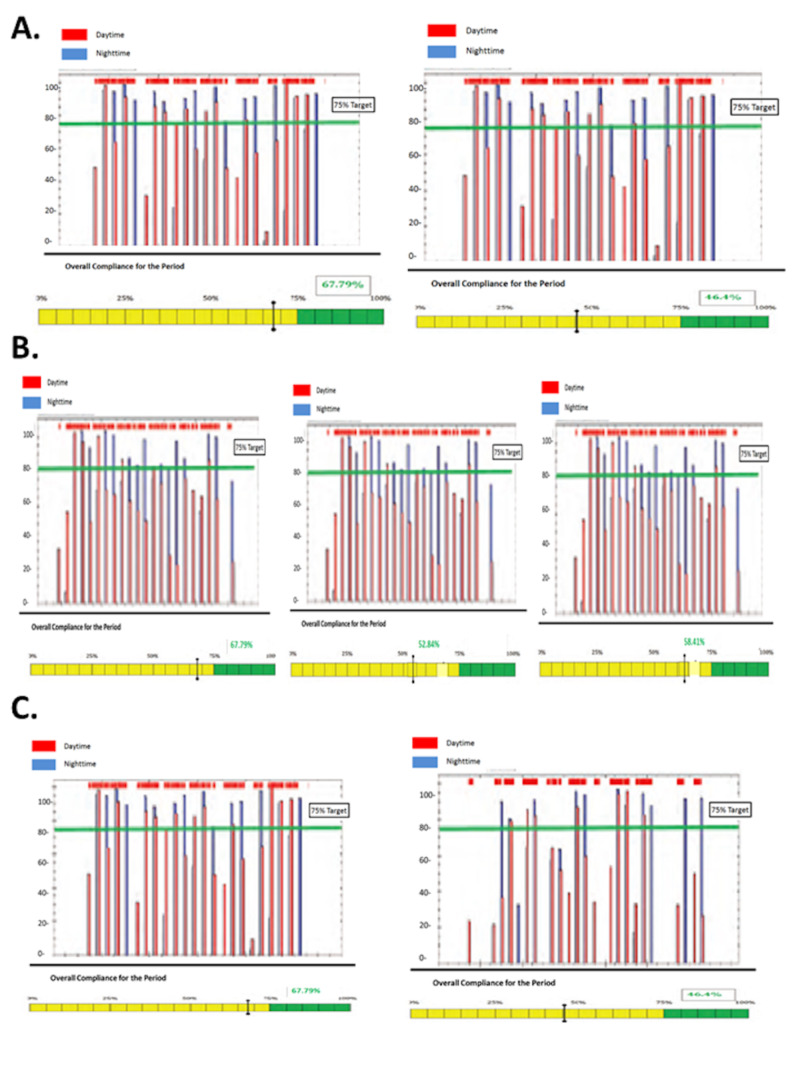
Tumor treating fields (TTFields) Compliance of Pediatric Patients Compliance rates are illustrated for patients 1-3 with a target of 75%.

A 15-year-old girl with a history of high-risk, pre-B cell acute lymphoblastic leukemia that was treated with multimodality chemotherapy and whole-brain photon irradiation (1800 cGy) presented two years after completion of therapy with behavioral changes. Magnetic resonance imaging (MRI) revealed a large left frontal-temporal neoplasm. The patient underwent gross total resection and the pathology was consistent with a diagnosis of GBM. Her treatment included oral daily TMZ and focal intensity-modulated radiation (photon) therapy. After completion of radiation therapy, maintenance treatment was started with maintenance TMZ and TTFields. Treatment with TTFields was well tolerated and was not associated with device-related side effects. Over the course of TTFields treatment, the average wear compliance rate for the patient was between 47.4% and 67.8% (Figure 1A). Two months after initiation of TTFields therapy, MRI showed disease progression and she died from her disease two months later.

Case 2

A 9-year-old boy presented with a several-month history of vomiting and headache, previously diagnosed as sinusitis. An MRI scan revealed a large posterior parietal neoplasm spanning the corpus callosum, without evidence of leptomeningeal metastatic disease.

Subtotal surgical resection was undertaken and the pathology was consistent with GBM. The patient subsequently underwent photon radiation with concomitant daily TMZ until progression four months after initial diagnosis. At this time, he was treated with TTFields in combination with TMZ, everolimus, and bevacizumab. No device-related toxicities were evident during the duration of TTFields therapy. An average wear compliance rates ranged from 52.2%-68.9% (Figure 1B). Three months after initiation of TTFields therapy, MRI showed progression and the patient subsequently died two months later.

Case 3

A 4-year-old boy presented with a three-week history of fatigue and progressive encephalopathy. An MRI scan revealed a large contrast-enhancing tumor localized to the left frontal temporal lobe. Following subtotal resection of the tumor that revealed a GBM, the patient failed treatments with carboplatin, vincristine, and TMZ and later focal photon radiation therapy, bevacizumab, temozolomide, irinotecan, and vorinostat. The patient began treatment with TTFields 14 months after initial diagnosis, which was continued for two months without evidence of device-related toxicity. The average wear compliance rate ranged from 91.2% to 92.1% (Figure 1C). The patient died of disease three months after initiation of TTFields.

Case 4

A 16-year-old male patient with a known germline p53 mutation, identified by TP53 gene-sequencing analysis after his mother died of leiomyosarcoma and his brother was treated for rhabdomyosarcoma, presented with a new onset seizure. MRI showed multifocal white matter signal abnormalities in the right occipital/parietal/temporal lobes with extension on T2/fluid attenuated inversion recovery (FLAIR) hyperintensity through the splenium of the corpus callosum. Biopsy revealed an anaplastic astrocytoma histology with a pattern of gliomatosis cerebri on neuroimaging.

His treatment prior therapies included radiation therapy, TMZ, bevacizumab, and a programmed death receptor ligand 1 (PDL-1) inhibitor as part of a clinical trial. Thirteen months after diagnosis, he was treated with TTFields in combination with daily TMZ. Therapy was well tolerated with no side-effects; specifically, no worsening of headache or skin irritation were reported. Unfortunately, no information on wear compliance was available for this patient. TTFields therapy was discontinued three months after initiation of therapy secondary to progression and the patient died of disease six months later.

## Discussion

The efficacy and safety of TTFields have not been extensively investigated in the pediatric brain tumor population and few data are available almost five years since the seminal adult clinical trial demonstrating its efficacy in adults with GBM multiforme [11]. Our case series adds to the previously reported five pediatric patients treated with TTFields [13-14] regarding the safety and tolerability. Our series describes two of the youngest patients ever to be reported with TTFields treatment.

Over the treatment periods of up to four months, adjuvant therapy with TTFields was well tolerated in these heavily pre-treated pediatric cases, and there were no device-related toxicities. These results are consistent with other recently reported cases [12-13]. Although limited conclusions can be drawn from this experience in children with heavily treated HGG, these preliminary safety observations in pediatric patients are consistent with the safety profile of adjuvant TTFields in adult GBM populations [11-12]. Local skin reactions have been reported with TTFields as a result of long-term wear [15-16]. None of our patients had any significant skin reactions.

As reported in a post-hoc analysis of the EF-11 trial of TTFields versus best clinical practice in adults with recurrent GBM, significantly higher survival was reported in patients with a wear compliance rate of ≥75% versus <75% (median OS 7.7 vs 4.5 months; P=0.042 [17]. Moreover, survival duration increased with increasing compliance [17]. Among the four pediatric cases presented here, the three-week average wear compliance ranged from 53% to 92% (median 68%) during the treatment periods, with night use of TTFields generally higher than day use. The compliance rate among this small group of pediatric patients is encouraging given positive associations between OS and higher wear compliance. It is unknown if outcomes would have been affected by improved compliance.

Our findings should be viewed with significant limitations aside from the retrospective nature and small number of patients. Our patients were very heavily pretreated and the use of TTFields was utilized many months after completion of standard radiation therapy. Furthermore, the compliance data was only available for three of four patients. All patients were treated off-label and outside of a clinical trial setting; no meaningful conclusions regarding efficacy can be determined. A clinical trial testing the feasibility of TTFields in children with recurrent or progressive supratentorial HGG and ependymoma is ongoing (NCT03598244).

## Conclusions

Our preliminary findings suggest that children with HGGs can tolerate TTFields for long durations per day and for treatment periods of up to four months without device-related toxicity. Feasibility studies of TTFields in pediatric patients with HGG in the clinical trial setting are ongoing.
